# Machine learning methodologies versus cardiovascular risk scores, in predicting disease risk

**DOI:** 10.1186/s12874-018-0644-1

**Published:** 2018-12-29

**Authors:** Alexandros C. Dimopoulos, Mara Nikolaidou, Francisco Félix Caballero, Worrawat Engchuan, Albert Sanchez-Niubo, Holger Arndt, José Luis Ayuso-Mateos, Josep Maria Haro, Somnath Chatterji, Ekavi N. Georgousopoulou, Christos Pitsavos, Demosthenes B. Panagiotakos

**Affiliations:** 1 Department of Nutrition and Dietetics, School of Health Science and Education, Harokopio University, Athens, Greece; 2 Department of Informatics & Telematics, School of Digital Technology, Harokopio University, Athens, Greece; 3 Department of Preventive Medicine and Public Health, Universidad Autónoma de Madrid, Madrid, Spain; 4 CIBER of Epidemiology and Public Health, Madrid, Spain; 5 Hospital Universitario de La Princesa, Instituto de Investigación Sanitaria Princesa (IP), Madrid, Spain; 6 The Centre for Applied Genomics, Genetics and Genome Biology, The Hospital for Sick Children, Toronto, ON Canada; 7 Parc Sanitari Sant Joan de Déu, Barcelona, Spain; 8 SPRING TECHNO GMBH & Co. KG, Bremen, Germany; 9 Health Metrics and Measurement, World Health Organization, Geneva, Switzerland; 10 Faculty of Health, University of Canberra, Canberra, ACT Australia; 11 School of Medicine, University of Athens, Athens, Greece; 12 CIBER of Mental Health, Madrid, Spain

**Keywords:** Cardiovascular disease, Risk prediction, Machine learning, Model performance

## Abstract

**Background:**

The use of Cardiovascular Disease (CVD) risk estimation scores in primary prevention has long been established. However, their performance still remains a matter of concern. The aim of this study was to explore the potential of using ML methodologies on CVD prediction, especially compared to established risk tool, the HellenicSCORE.

**Methods:**

Data from the ATTICA prospective study (*n* = 2020 adults), enrolled during 2001–02 and followed-up in 2011–12 were used. Three different machine-learning classifiers (k-NN, random forest, and decision tree) were trained and evaluated against 10-year CVD incidence, in comparison with the HellenicSCORE tool (a calibration of the ESC SCORE). Training datasets, consisting from 16 variables to only 5 variables, were chosen, with or without bootstrapping, in an attempt to achieve the best overall performance for the machine learning classifiers.

**Results:**

Depending on the classifier and the training dataset the outcome varied in efficiency but was comparable between the two methodological approaches. In particular, the HellenicSCORE showed accuracy 85%, specificity 20%, sensitivity 97%, positive predictive value 87%, and negative predictive value 58%, whereas for the machine learning methodologies, accuracy ranged from 65 to 84%, specificity from 46 to 56%, sensitivity from 67 to 89%, positive predictive value from 89 to 91%, and negative predictive value from 24 to 45%; random forest gave the best results, while the k-NN gave the poorest results.

**Conclusions:**

The alternative approach of machine learning classification produced results comparable to that of risk prediction scores and, thus, it can be used as a method of CVD prediction, taking into consideration the advantages that machine learning methodologies may offer.

## Background

Developed and developing countries have succeeded in protecting their populations from infectious and parasitic diseases through structured health systems and preventive campaigns including vaccination policies and regular health examinations [1, 2]. Interestingly, while communicable diseases’ incidences have steeply decreased during the past century, non-communicable diseases, such as cardiovascular disease (CVD) and malignancies have been both the direct and the underlying cause for the majority of deaths [3]. Although CVD mortality rates are currently declining in most European countries [4], there is an increasing non-fatal CVD incidence, especially among females and younger individuals, leading also to an increasing financial and social cost [5, 6]. However, CVD is a highly avertable health condition that can be prevented, delayed or even well controlled when it is diagnosed at early stages of atherosclerosis process, through a number of lifestyle changes and accurate pharmaceutical treatment and management. Under this context, an emerging need to better and early identify high-risk individuals is highlighted as a first priority in order to reduce the burden of CVD, allowing more effective intervention and thus more disease-free years [7].

The use of CVD risk estimation scores (or tools) was initially suggested in the Framingham study [8], which was used to predict individual CVD risk in US, but also in many other countries around the world, using low-cost variables, such as age, gender, smoking habit, cholesterol and blood pressure levels, etc. [8]. In the early 2000s Menotti et al., [9] revealed some methodological drawbacks of the Framingham CVD risk score when applied to different populations around the world, using the Seven Countries Study dataset. Similar considerations have also arisen from various groups regarding the accuracy of a health risk prediction tool when applied to different population from the one that was developed. Under this context, in early 2000s, the European Society of Cardiology (ESC) established the SCORE project as an attempt to develop a more accurate risk prediction tool for the European populations [10]. Since then, the SCORE is being used across European countries whilst many of these countries are using population-specific calibrated models in order to achieve the best individuals risk predictions. Among them, one of the pioneer countries was Greece which has recalibrated the European Society of Cardiology (ESC) SCORE into the HellenicSCORE by considering the prevalence of CVD risk factors in the Greek population [11]. It should be noted here - for the reader who is not familiar with CVD risk prediction scores - that there is a variety of CVD risk prediction tools, from different countries and populations, with different set of risk factors used and with a large variation regarding their performance. The majority of these scores use a common set of the “classical” CVD risk factors, e.g., age, sex, smoking, blood pressure and lipids levels, whereas others have also incorporated more advanced markers of CVD disease. The methodological framework of the vast majority of these risk prediction tools is based on stochastic - statistical - models that incorporate individual variables, based on cohort studies, in order to calculate overall risk for a future event [12]. Despite the aforementioned approaches to early identify the potential CVD candidate through risk prediction tools, a high percent of CVD events occurs in people without established risk factors, or with low-to-moderate overall risk, whereas, approximately 20% of high-risk individuals, remain underestimated due to risk misclassification, suggesting the need to identify new methodologies that could optimize the performance of risk prediction [13–16].

Due to the large amount of available data that requires analytical processing, a category of algorithms for data manipulation that has been introduced in various scientific fields, including health, is that of machine learning (ML). ML is a sub-area of artificial intelligence, with an ultimate goal to devise learning algorithms that do the learning automatically from the available data with minimal or even none human intervention. This area comprises numerous different types of algorithms capable of processing large amounts of data and that ultimately transform data into knowledge, which can be used to infer some intelligent action or decision. The interest in ML in health sciences has grown since the early 2000s [17] and ML has been applied in various healthcare and biomedicine applications [18], including cancer prognosis and prediction [19], radiologic imaging [20], the understanding of ageing process [21] and of course CVD risk prediction [22]. ML, similarly to well-known and established statistical approaches, aims at “learning” from data. In the statistical approach, mainly a probabilistic model is built, based on the assumption that the provided data are a subset of a larger population that can be described by a model. In principal, a simpler model is much more preferable than a complex one, as long as there is an acceptable performance. Moreover, human intervention is considered essential in every stage of the overall build of the model [23]. On the other hand, ML emphasizes more on predictions and thus the efficiency is evaluated via prediction performance.

The CVD risk estimation is clearly a typical classification problem, where an individual must be somehow categorized as having a low or a high CVD risk. As it will be shown in the following, it is possible to correctly classify an individual to an actual CVD risk class, using ML techniques based on various easily accessible data regarding individual’s bio-clinical risk factors, socio-economic, lifestyle and psychological characteristics. Established risk prediction tools, such as the HellenicSCORE, induct their prediction based on a very limited number of CVD risk factors that can be easily evaluated in daily practice. On the contrary, ML techniques exploit the majority of the available data, building much more complex models considering many more features than only the typical CVD risk factors. In the order to go forward with the comparisons, among the vast variety of available ML algorithms, three well-known and established ML algorithms were chosen: a) One of the simplest classifiers, the k-nearest neighbors’ algorithm (k-NN) [24], which is yet quite efficient in general, b) A more complex one, the Quinlan’s C5.0 decision tree algorithm [25] which uses a tree structure to locate the connections among the data, making the decision process quite transparent and informative, and c) A quite complex meta-learning algorithm, that of random forest (RF) [26].

**Fig. 1:**
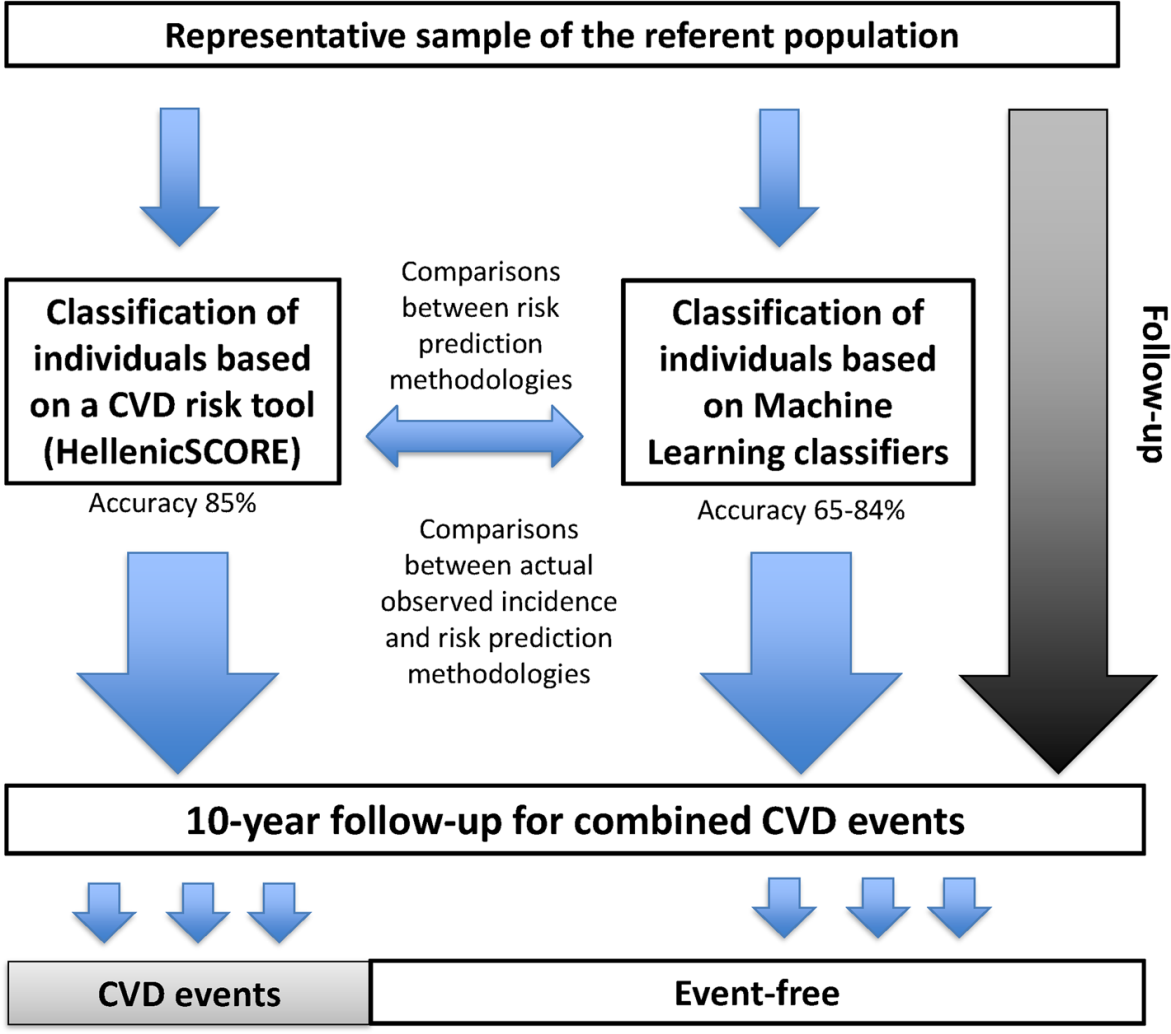
Conceptual methodology applied in the present work to evaluate two approaches for CVD risk classification, a classical, statistically oriented risk prediction tool and machine learning algorithms

Thus, the aim of the present work was to explore the potential of using ML methodologies on total cardiometabolic risk assessment of healthy adults. Therefore, the predictive accuracy of ML methodologies was compared to already known and established risk prediction tools, the HellenicSCORE, a calibration of the ESC SCORE, against the 10-year combined (fatal or non-fatal) CVD incidence; the research hypothesis was to evaluate which approach for risk classification improves the correct class CVD prediction rate of the referent population. Figure 1 illustrates the methodological framework of the present study, in a high-level approach.

## Methods

### The ATTICA study cohort

The working dataset was based on the ATTICA prospective cohort study, performed in the Athens metropolitan area, Greece. During 2001–2002, *n* = 3042 Greek adults (1528 women and 1514 men), stratified by age-sex category of the Greek population (according to 2001 census), were enrolled on a volunteer basis [27]. A large amount of information was collected, and participants were followed up in 2011–12 [28, 29]. Out of the 2583 individuals that participated in the 10-year follow-up, *n* = 2020 participants had data without any missing information regarding the development of a combined (fatal or nonfatal) CVD event (coronary heart disease, angina, heart failure or stroke, according to ICD-9 classification). The combined 10-year CVD incidence was 15.7% (19.7% in men and 11.7% in women, *p* for gender difference < 0.001). Table 1 illustrates baseline characteristics of the *n* = 2020 participants that were used as a sample for the development of ML models.

**Table 1:** Description of the dataset containing the 16 baseline variables that were measured among *n* = 2020 ATTICA study participants

Variable used in ML	Male	Female
Age in years, mean ± SD	46 ± 13	45 ± 14
Smoking status at baseline, %(yes)	44%	37%
Years of school mean ± SD	12.3 ± 3.6	12.0 ± 3.8
MedDietScore (range 0–55), mean ± SD	24 ± 5	27 ± 7
Basic metabolic rate as a proxy of energy expenditure	1783 ± 228	1384 ± 128
Body mass index in kg/m^2^, mean ± SD	27.3 ± 3.9	25.2 ± 4.7
Diastolic blood pressure levels in mmHg, mean ± SD	82 ± 11	76 ± 11
Systolic blood pressure levels in mmHg, mean ± SD	127 ± 17	118 ± 18
History of hypertension (including medication), %	39%	24%
Glucose levels (in mg/dl), mean ± SD	95 ± 25	90 ± 22
History of diabetes mellitus (including medication), %	8%	6%
Total cholesterol levels (in mg/dl), mean ± SD	197 ± 42	191 ± 41
Triglycerides (in mg/dl), mean ± SD	140 ± 102	98 ± 56
History of hypercholesterolemia (including medication), %	46%	38%
Interleukin-6 levels (ng/ml), mean ± SD	1.5 ± 0.5	1.4 ± 0.5

The initial ATTICA study dataset underwent an intense preprocessing phase before being used by the ML classifiers. In a first step, from the dataset of 100 bio-clinical and lifestyle behavior variables that have long been associated with the development of CVD, 43 were selected on the bases of non-highly inter-correlated. This step was of crucial importance, since the existence of numerous variables in a dataset may render the model very complex and most probably over-fitted to the dataset. In the next step, all variables with missing values in more than 70% of the individuals were excluded. For some of the remaining 22 variables, there were some levels of missing data (< 10% of the total sample), which were replaced by imputed data, using the common approach of KNN imputation [30] based on 10 neighbors. Moreover, a student’s *t*-test was applied to compare each variable separately with the individual’s classification as low or high CVD risk; variables passing the chosen threshold of *p*-value 0.01 were excluded, keeping 16 variables (pls see Table 1). The variables were then tested for high inter-correlation, which could lead to a biased model towards the correlated variables; however, no variables were excluded in this final step (Pearson *r* correlation coefficients < 0.8). Hence, at the end of the preprocessing phase the working dataset contained 16 baseline variables, the ones shown in Table 1.

### Statistical approach for CVD risk assessment: the HellenicSCORE

The HellenicSCORE [31] is a calibration of the official ESC SCORE and was calculated based on age, sex, smoking status, systolic blood pressure and total cholesterol levels of the participants’ baseline characteristics. Specifically, based on (a) the risk factor prevalence that was obtained from the baseline evaluation of the ATTICA study in 2001–2002 [12, 27], (b) the annual death rates that were obtained from the World Health Organization mortality database for 2002 [32] accordance to the rules of the International Classification of Diseases (ICD) [33], and (c) the individuals’ characteristics, a recalibration method for the Greek population was applied separately for men and women, and the estimated 10-year risk for fatal CVD events was calculated for each participant using Cox Proportional Hazard models [12].

### Machine learning methodologies for CVD risk assessment

Three different ML classifiers were chosen and hence, three distinct models were created in order to distinct whether an individual had a low or a high CVD risk. Obviously, the list of tested algorithms is not exhaustive, other methods such as Naive Bayes [34] or Support Vector Machines [35] could also be used, but preliminary tests showed that the chosen classifiers give satisfying results, with regards to missing data, outliers and computational time. Another technique for implementing ML, very well established and currently very popular, is that of deep learning [36]. However, deep learning is typically applied when there is a very large amount of available data, which is not the case in our dataset since it consists of around 2000 participants. For cases where the available dataset is relatively small, traditional machine learning techniques prevail and thus, deep learning techniques were not considered.

ML techniques in general are applied in two stages: at first, a (random) subset of the available data (called training set) is used for the training of the model and next, the rest of the data (called testing set) are used for the evaluation of the model. The training set is used to induce a model, capable of predicting the annotations of the instances in the testing set. The choice of each subset’s elements is of major importance and must be representative of the original dataset, i.e. the sampling must be completely random and not biased in any way. A common practice is to use a stratified k-fold cross-validation (k-fold CV), where the data are partitioned into k segments of equal size (folds) which retain the ratio between classes, and k independent iterations of training/validation are performed.

**Fig. 2:**
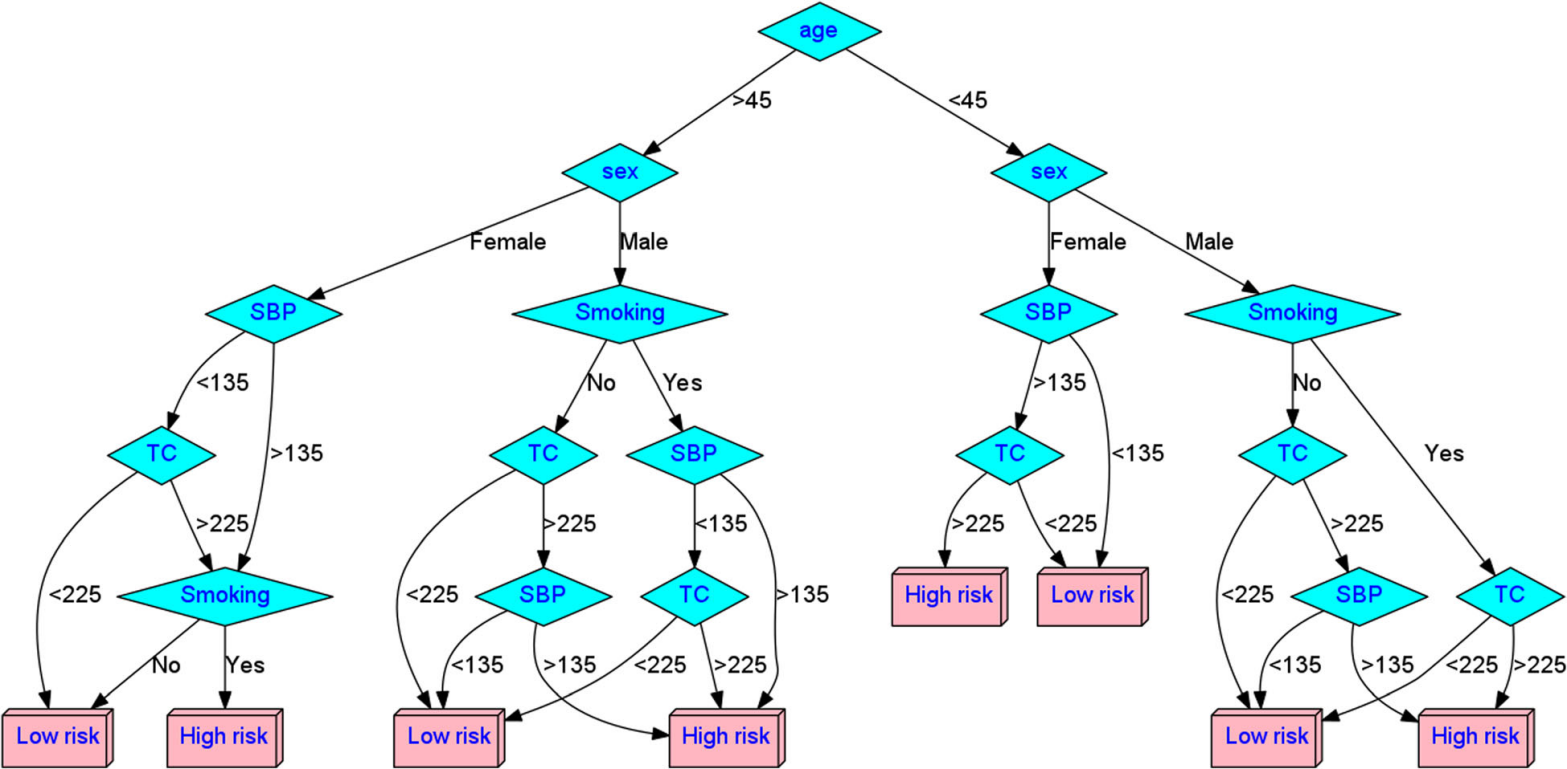
An example of a Decision Tree (DT) derived using the 5-variable dataset (age, sex, systolic blood pressure levels (SBP), total cholesterol (TC), and smoking). The thresholds for the quantitative variables (Age, TC, SBP) derived from the algorithm used to develop the DT

As already mentioned, the three ML algorithms chosen were k-NN, DT, and RF. The k-NN is one of the simplest classifiers but yet quite efficient in general. The data are clustered into similarity groups using k “neighbors” for the classification of each individual. The main principle is that instances within a dataset will generally exist in close proximity to other instances that have similar properties [37]. Once the instances are binned into classes, then an unclassified instance can be classified by observing the class of its nearest neighbors. DT is a classifier, which uses a tree structure to locate the connections among the data, making the decision process quite transparent and informative. The tree structure classifies instances based on feature values: each tree node covers a feature in an instance to be classified, and each branch represents a value that the node can assume. Starting from the root node, every instance is classified according to their feature values. Using such decision algorithms, a clinician may better visualize and classify an individual into risk categories for developing a disease. In general DTs make the classification process more “human readable”, e.g. the decision tree produced by the DT algorithm shown in Fig. 2, does not need any expertise to be followed and thus categorize an individual. RF is a tree-based ensemble classifier, which provides additional diversity in the created tree model; RF can also be seen as an adaptively weighted potential nearest neighbor [38] classifier. In general, ensemble methods create a stronger learner by combining multiple weaker learners. RF models are preferable to other tree-based classifiers, since they are less prone to over-fitting [39]; however, unlike decision trees, the rationale behind the produced model is not always easy to interpret. RF can cope with datasets with extremely large number of features since only the most important features are considered by the algorithm.

Before starting the training of the ML models, some additional preprocessing was necessary. The k-NN clustering is using as metric the Euclidean distance between the individuals; however, the quantitative variables of the dataset had different ranges, so in order for each variable to have the same impact on the distance, all numeric variables were normalized into the range of [0,1].

### Comparisons between ML approaches vs. HellenicSCORE on 10-year CVD risk

Using the three ML techniques every individual was classified into one of the two disjoint categories of “low” (< 10%) and “high” (> 10%) 10-year CVD risk - following also the rationale of the ESC classification. These classifications were tested against the same classifications resulting from the HellenicSCORE prediction tool, i.e., HellenicSCORE < 10% (*n* = 1912 individuals, 95% of the sample) and > 10% (*n* = 108 individuals, 5% of the sample). Moreover, according to the observed 10-year CVD incidence of ATTICA study, individuals were classified as CVD-free (*n* = 1707, 84%) and CVD incident cases (*n* = 317, 16%). The three ML techniques were trained and evaluated using different sets of the preprocessed data, in an attempt to achieve the best performance. Specifically, the first set used included all the 16 available variables of the preprocessed data mentioned above, that represent widely accepted CVD risk factors. The second set contained only the 5 variables also used for the calculation of the HellenicSCORE (i.e., age, sex, smoking, total cholesterol, and systolic blood pressure levels). The latter set was used in an attempt to explore the performance of the ML models using only the data available to the HellenicSCORE, i.e. comparing the performance of the two different approaches using the same input data. While the first set was used in an attempt to build a more complex model, exploiting additional variables disregarded from the HellenicSCORE. Additionally, since the different percentages of disease-free individuals compared to cases inevitably leads to biased models towards the healthy individuals, to overcome this bias, two additional training datasets were created, using bootstrapping techniques on both the 16 variables dataset and the 5 variables dataset.

Hence, to recap, five different comparisons were made:

1. *ML_16_vs_HS*: using as input the 16 variables, the results were compared against the HellenicSCORE.
2. *ML_16_vs_CVD*: using as input the 16 variables, the results were compared against the 10-year CVD incidence of ATTICA study participants.
3. *ML_16_Boot_vs_CVD*: based on the 16 variables and using bootstrap techniques a more balanced training dataset was created and compared against the 10-year CVD incidence.
4. *ML_5_vs_CVD*: using as input the 5 variables, the results were compared against the 10-year CVD incidence.
5. *ML_5_Boot_vs_CVD*: based on the 5 variables and using bootstrap techniques a more balanced training dataset was created and compared against the 10-year CVD incidence.

Once the dataset was normalized, 10 different random folds were created, each containing the 10% of the dataset. Each fold was then divided into two disjoint sets; one containing 90% of the fold data, used for the building of the k-NN model and the rest 10% for the evaluation of the produced model. The chosen number of neighbors to be considered in the construction of the model was set to 3. No data normalization was needed for the RF classifier. Regarding the folds, similarly to the k-NN approach, the same 10 different random folds were used. For each fold, an ensemble of trees was created with each one having one vote and the model decided on the classification of each individual using the majority rule. The number of trees grown in every fold in the construction of the model was set to 35. For the case of the DT classifier, there was also no need for the normalization of the variables. In order for the results to be comparable with the two aforementioned methods, the same 10 different random folds were used. Regardless of the chosen model, once it had been trained there was a need to evaluate it against known data. For the evaluation of each algorithm, the number of true positive (TP) and false negative (FN) were counted for the individuals predicted as high CVD risk. Similarly, of the individuals predicted as low CVD risk, the number of true negative (TN) and false negative positive (FN) were counted. Based on these values, five metrics were calculated for each algorithm, to evaluate the diagnostic accuracy, i.e. the amount of agreement between the results from a model and those from the known data. Namely:

- *Accuracy*: the ratio of the correctly classified individuals to the total number of individuals, i.e. $\frac{TP+ TN}{TP+ TN+ FP+ FN}$_._
- *Sensitivity*: the probability of predicting the individual’s class as CVD risk hazardous when it truly is CVD risk hazardous, i.e. $\frac{TP}{TP+ FN}$.
- *Specificity*: the probability of predicting the individual’s class as non-CVD risk hazardous when it truly is non-CVD risk hazardous, i.e. $\frac{TN}{TN+ FP}$.
- *Positive Predictive Value (PPV)*: the probability the individual is CVD risk hazardous when it is predicted as such, i.e. $\frac{TP}{TP+ FP}$.
- *Negative Predictive Value (NPV)*: the probability the individual is non-CVD risk hazardous when it is predicted as such, i.e. $\frac{TN}{TN+ FN}$.

Neither of these metrics alone can absolutely characterize the performance of a model, e.g. a high sensitivity alone does not make a model a good model; it needs to also recognize all individuals with low CVD risk, i.e. specificity. However, in a daily practice, an individual will be more interested in the PPV and NPV, i.e. the probability of being high CVD risk after such a classification by the ML model. Whereas the NPV is the probability of being low CVD risk after such a classification by the ML model. All these metrics have different pros and cons, and they may be difficult to unilaterally interpret [40]. Therefore, one sometimes prefers a combination of them. For all the conducted types of comparisons, regardless of the dataset and the ground truth labels used for the evaluation, the same methodology was applied to each ML technique. For each of the classifiers, once all 10 models had been produced and evaluated, the average performance across all models was calculated and assigned as the total performance of the produced model. All the implementations were carried out in the R programming language [41] (version 3.3.3). For the k-NN classifier the *class* [24] R-package was used, for the DT classifier the *C50* [25] R-package, and for the RF the *randomForest* [26] R-package. It is noted that the process of creating the folds and using each different fold to train and evaluate the classifier is computationally intensive, but the procedure is completely independent for each fold and thus it is possible to parallelize it by assigning each fold to a different computer core. Using in-house R scripts, the execution was parallelized into different computer cores, achieving a speed-up almost equal to the number of computer cores used for the calculations.

## Results

All the possible combinations of the five different comparisons mentioned in the Validation subsection and the three different ML classifiers were performed, in order to evaluate the performance of the ML. The performance metrics - sensitivity, specificity, NPV, PPV and accuracy - of either the HellenicSCORE or the ML classifiers on the observed 10-year CVD incidence, as well as the between ML classifiers and HellenicSCORE classification, is presented in Tables 2, 3, 4, 5 and 6. In Table 2 the first comparison *(ML_16_vs_HS)* is presented, where as it can be seen all three ML methods had higher performance as compared to HellenicSCORE classification, achieving accuracy rates of 96, 99, and 99% for the case of k-NN, RF, and DT classifier, respectively. The same stands for the sensitivity rates, which were 98% for the k-NN, 100% for the RF, and 99% for the DT classifier. However, DT outperformed all other methods regarding specificity with a rate of 87% compared to 37 and 79% for the k-NN and RF classifiers. Regarding the PPV, the lowest performance was 97% for the k-NN and the highest 99% for the other two classifiers; however, the NPV was 79% for k-NN, 89% for the DT and 98% for the RF. In total, based on all five metrics the RF classifier achieved the best performance, DT the second best, while k-NN had the poorest performance. The specific comparison certified that the ML techniques - especially RF and DT - had comparable efficiency one another and superiority to that of HellenicSCORE.

**Table 2:** Performance of the three ML algorithms using the 16-variable dataset against the predicted 10-year CVD risk through the HellenicSCORE

Algorithm	Accuracy	Specificity	Sensitivity	PPV	NPV
k-NN	0.96	0.37	0.98	0.97	0.50
RF	0.99	0.79	1.00	0.99	0.98
DT	0.99	0.87	0.99	0.99	0.89

**Table 3:** Performance of the three ML algorithms using the 16-variable dataset and of the HellenicSCORE, against the 10-year CVD (fatal or non-fatal) incidence of ATTICA study participants

Algorithm	Accuracy	Specificity	Sensitivity	PPV	NPV
k-NN	0.83	0.24	0.94	0.87	0.47
RF	0.84	0.20	0.96	0.87	0.46
DT	0.84	0.17	0.96	0.86	0.42
HellenicSCORE	0.85	0.20	0.97	0.87	0.58

**Table 4:** Performance of the three ML algorithms using the 16-variable dataset with bootstrapping and of the HellenicSCORE, against the 10-year CVD (fatal or non-fatal) incidence of ATTICA study participants

Algorithm	Accuracy	Specificity	Sensitivity	PPV	NPV
k-NN	0.65	0.56	0.67	0.89	0.24
RF	0.83	0.46	0.89	0.90	0.45
DT	0.80	0.53	0.85	0.91	0.40
HellenicSCORE	0.85	0.20	0.97	0.87	0.58

**Table 5:** Performance of the three ML algorithms using the 5 variables dataset and of the HellenicSCORE, against the 10-year CVD (fatal or non-fatal) incidence of ATTICA study participants

Algorithm	Accuracy	Specificity	Sensitivity	PPV	NPV
k-NN	0.82	0.21	0.93	0.86	0.35
RF	0.84	0.22	0.95	0.87	0.45
DT	0.84	0.14	0.97	0.86	0.49
HellenicSCORE	0.85	0.20	0.97	0.87	0.58

**Table 6:** Performance of the three ML algorithms using the 5 variables dataset with bootstrapping and of the HellenicSCORE, against the 10-year CVD incidence of ATTICA study participants

Algorithm	Accuracy	Specificity	Sensitivity	PPV	NPV
k-NN	0.66	0.62	0.67	0.91	0.26
RF	0.79	0.47	0.85	0.90	0.37
DT	0.78	0.48	0.84	0.90	0.36
HellenicSCORE	0.85	0.20	0.97	0.87	0.58

For the rest of the comparisons, the evaluation was measured against the observed 10-year CVD incidence. In Table 3 the results from all the models built based on the 16 variables *(ML_16_vs_CVD)* are presented, while in Table 4 the training dataset *(ML_16_Boot_vs_CVD)* as created using bootstrapping. It is clear that the accuracy decreased slightly when bootstrapping was used; however, the specificity increased more than double its value by the usage of bootstrapping. This happened, since the ML models were better trained to recognize unhealthy cases in the test dataset. Regarding the ML models, once more the RF one outperformed the one of DT, especially in the bootstrap case. Indeed, the results of the ML methods were completely comparable to the ones of HellenicSCORE and performed better regarding specificity.

Similarly, in Table 5 the results are presented for the models built using only the 5 variables also used for the calculation of the HellenicSCORE *(ML_5_vs_CVD)* and in Table 6 the same dataset *(ML_5_Boot_vs_CVD)* was created using bootstrapping. The accuracy when no bootstrapping was used was similar to that of the model built with the 16 bootstrapping; however, the specificity was less than half of it. When bootstrapping was used, the specificity almost doubled, but still the accuracy and the sensitivity decreased.

Therefore, in conclusion the better ML performance was achieved when more variables were used for the training of the models and the training was conducted on bootstrapped dataset, i.e., when all 16 variables were used and not only the 5 factors used for the calculation of the HellenicSCORE. Since the performance metrics for all classifiers were the mean values of respective metrics for the 10 folds, variances for all metrics per method were calculated (data not shown). The variances were small, meaning that the produced models of each fold had similar efficiency and the data used in each fold were homogenous with no significant outliers.

## Discussion

Accurate risk prediction is a cornerstone in public health care. Several risk prediction scores or tools have been proposed that past years to identify the potential candidate for developing a CVD or cancer event. However, their accuracy in correcting classifying a candidate for developing the disease, is doubtable. Thus, the use of risk tools at population level, although of substantial clinical value, has not been well appreciated. In this work we introduced ML methodologies in predicting CVD events and compared their classification with an already established and used risk prediction tool. According to the presented results, the application of ML approaches in CVD risk prediction may further assist in correctly identifying individuals at high risk and in applying more effective population-based strategies. Regarding the specificity and the NPV, the results were more satisfying when bootstrapping was applied for the creation of the training dataset. Indeed, bootstrapping techniques lead to a less biased model toward the healthy individuals, since the training sets are generating from the original dataset with replacement, and thus making them balanced by including more well-adjusted number of healthy and unhealthy individuals. However, regarding the k-NN classifier, especially for the case that a larger set of variables was used combined with bootstrapping, the results for specificity and sensitivity, as well as the NPV, were not so promising. Some reasons for that might be the inherent outlier sensitivity of the specific algorithm, combined with the large dimensionality of the used data; k-NN is known to suffer sometimes from the Bias-Variance tradeoff [42], i.e., it can become over-fitted to the training data and perform poorly on the testing data.

At this point it could be argued that the ML approaches are more complicated, compared to the classical risk prediction tools. However, nowadays, where information technology has become a “daily” use practice in clinical setting, the development of ML-based tools where a clinician can easily impute with basic and simple individuals’ characteristics and correctly calculate their future risk for CVD or any other disease, would be of considerable importance for the public health. Moreover, although ML may be more complicated than the common risk prediction tools, a main advantage of the ML approach is their inherent ability to evolve large sets of variables, based on the accumulated data, i.e., the more data that become available, the better model is built without any a-priori defined methodological restrictions. In addition, and in contrary to the classical risk prediction tools, simply repeating the training phase of the classifier, using the accumulated data, does the building of a new model, more accurate, which is a cornerstone in public health care setting. Additionally, the unbiased view of the available data by the ML algorithms, can lead to the discovery of previously unseen relationships among data, which were very often present, but ML allowed them to visualize, whereas the classical approaches may not due to models restrictions. However, one may claim that the use of more variables to build the ML model than the risk prediction tool (i.e., 16 vs. 5 variables) lead to a a-priori defined superiority of the ML model due the higher information used. The use of a relatively small set of variables in risk prediction tools is common in practice because of two main reasons: the need to be the risk classification as simple as possible in terms of general public health use, but also the pre-requirement of the models not to be parsimonious and to reduce the level of undesirable colinearity. Nevertheless, ML models performed similar compared to the risk prediction tools even when the same set of variables used. Therefore, although the results of comparison between ML and classical approaches were comparable, in the near future the abundance of available data and the discovery of unseen so far relations, will render ML a very powerful tool for the life scientists in the CVD risk prediction.

In ML applications due to the larger number of variables typically taken under consideration compared to classical approaches, the created ML model is often considered as a “black box”, since the model’s details are too complex to be presented to the end user. The models created by the k-NN and RF classifiers fall into the latter category. Although they have been evaluated and new individuals can be classified, the rationale behind the decision to eventually categorize one individual into one category or the other is not transparent. This happens in the attempt to hide the complex mathematics models implemented, based on the training dataset. This necessary issue is common knowledge in the area of ML, however, especially for health issues the patient wants to be able to know the reasons she or he was categorized into one class over the other. On the contrary, the decision tree created by the DT classifier is much more straightforward to understand, without special knowledge. This simplicity in the model representation often comes with the cost of the model being more “naive”; indeed, the DT classifier has a lower performance compared to the RF one. As a limitation, it must be noted that the current study like other similar studies [20, 43, 44] was based on a cohort of small to medium size. Ideally, the same methodology would be replicated using data of a much larger cohort, in order to further support the findings. Additionally, the highly disproportional percentages of individuals having high CVD risk compared to those with low risk inevitably leads to biased models towards the individuals with low risk, i.e., the models built are better at correctly identifying low CVD risk individuals compared to high risk ones. This can be seen on all three models, where the sensitivity was much higher than the specificity; in other words, the model produces relatively more FP than FN, since it can recognize easier a low risk individual over a high risk one. The latter was handled by using bootstrapped data for the training of the ML models and thus artificially balancing the abundance of healthy individuals compared to CVD ones.

## Conclusions

This study explored the potential of applying ML approaches on population data, alternatively to the established risk prediction tools. The results showed that ML performs comparable well with the established risk tools in identifying a potential candidate for CVD development. In particular, three machine-learning classifiers were compared against an estimation tool for CVD risk prediction, as well as against actual CVD incidence, giving very high accuracy, sensitivity, and PPV for the classification. A main advantage of the ML approaches is their inherent ability to evolve large sets of variables, based on the accumulated data, i.e., the more data that become available, a better model is built without any a-priori defined methodological restrictions. In addition, and in contrary to the established approaches, simply repeating the training phase of the classifier, using the accumulated data, does the building of a new model, more accurate and automatic. Additionally, the unbiased view of the available data by the ML algorithms, can lead to the discovery of previously unseen relationships among data, which were always present, but ML allowed them to visualize, whereas the classical approach may not – mainly due to the constraint of colinearity. Therefore, although the results of comparison between ML and classical approaches were comparable, in the near future the abundance of available data and the discovery of unseen so far relations, will render ML a very powerful tool for the life scientists in the CVD risk prediction. To conclude, based on the presented results, the ML approach can prove a valuable and helpful methodology in the field of CVD risk prediction, and not only, making prognostication algorithms easier to understand and use in clinical practice. Indeed, in spite of the claims that ML classification techniques can result in adequate and effective decision making very few have actually penetrated the clinical practice [19]. As a future step, in the effort of establishing ML in the field of CVD prediction, larger datasets will be sought, which will allow to build more accurate models. Ideally, such datasets will contain individuals from various nationalities, making the built models international. Indeed, such datasets can be produced from the available databases of the currently ongoing ATHLOS (Ageing Trajectories of Health: Longitudinal Opportunities and Synergies) project. Once the various datasets are homogenized, numerous different ML models will be tested; given the large number of available individuals Deep Learning techniques could also be tested. Therefore, all the above will allow us to identify and understand relationships between CVD and various lifestyle characteristics yet undiscovered by the available tools.

## Abbreviations

CV: Cross-validation
CVD: Cardiovascular disease
DT: Decision tree
ESC: European Society of Cardiology
k-NN: k-nearest neighbors
ML: Machine learning
NPV: Negative predictive value
PPV: Positive predictive value
RF: Random forest
